# Supplementary material for: Patterns of Intron Gain and Loss in Fungi
Source: PLoS Biol. 2004 Nov 30;2(12):e422. doi: 10.1371/journal.pbio.0020422 (PMC532390; doi:10.1371/journal.pbio.0020422)
Supplement: Table S1 — Also available at http://genes.mit.edu/NielsenEtAl/. (4.3 MB ZIP). [file pbio.0020422.st001.zip › NielsenEtAl/html/780.html]

AN4557.1.NCU01479.1.MG07441.1.FG06816.1


```
 CLUSTAL W (1.82) Multiple Sequence Alignments - Introns Inserted


Sequence 1: MG07441.1	1009 aa
Sequence 2: FG06816.1	885 aa
Sequence 3: AN4557.1	883 aa
Sequence 4: NCU01479.1	928 aa
Alignment Length: 1041 aa
Number Identitical Residues: 377 aa
Alignment Score (without introns) 16553


MG07441.1 	MSRLLRLVPR-QLAAG-QSVPILLRASPRLPSLHAVRLPRTYRVGAVGSLRSFS-HNSRL
NCU01479.1	MAVRFRQSSQQLALCARQALRTTTRPAYRASRLPRASLSSARQLPTSRLYSTQSTEKPEP
FG06816.1 	MSRLLRQSSQ-FARVTRLSSQPALRLCAR-PSIIARSNPAKTSAAFAIRARTYS-SQP-P
AN4557.1  	MASLLRRPGN-LAQYSRRAAEHIFRARPLQTRISQSRLSSLYTAHRVRTFTSQRDVDPKP
          	*:  :*   .        :     *     . :     .            :    ..  

MG07441.1 	LSSQNPSEPPEKKD--GSEKSSQTPQS---PPSDAARSAEATELPLLEGHVRITKEEADS
NCU01479.1	KKDDDNKSPKPEEDEEDFEPKRPLPPGWVRMEKQDLKDFDAFIKKAALKTGIKIEGQDVI
FG06816.1 	PSGQDPRSNPDRDNEHGSKPLNQKKAD---DGVEGGKSEAPKTPPLPDGWIHLSKEEIAH
AN4557.1  	ER----RVPPTENDGKQDDGGNSQPP----QKPEGPKDSEEPNKPEEDK---KPEQPPSK
          	           ..:    .              :  :.                :     

MG07441.1 	LRDLA-----SDMDEAGRQNFHKWVGIWEQRGAPEVAKDFAQKHLGPG-LGFMDGIYLVD
NCU01479.1	LQGIKHYGCPKETRELLKKWISGNITIFEQLRCMKSLVMLAKDLGEHE-VEYMRKNPNAK
FG06816.1 	LEEFT-----SRLPEAQRPVAKDILQKLQVVGAPSETRDLLQKLRQNGNLSILDKGRLMR
AN4557.1  	LT--------AEEEEQVKQFFE-HMTQFFPASQINDLRIFVRILQKNG-MTPETRQFIEK
          	*             *  :      :         .    : :       :          

MG07441.1 	VLREHSKPPPEDLFVPQANETNDKSAPTTEKSHQSQTQERPESQDSQASSSPEDLVKGWV
NCU01479.1	SIASGFN---DNTFTSNMSDSDRKAQTSEEQNKQSEGKEAGQQQSDGQQGKQSGEQQGQQ
FG06816.1 	CVYQVT----ERIIDLEENQGQDGQFSSFRMDQESTSQDGKNGADK----SKQQQQKGGK
AN4557.1  	YIVTGK----EPSMMEYMRISTHVQYTVRKNASQENEQKDGQKEEQKQSGQNGQKQQGKS
          	 :        :  :            .  .   :.  :.  :  ..  ...     :*  

MG07441.1 	QISDEEKQAFLAIVEMLPKNQQAN-------MKAFAECAQKHGVPPAMKELIQKHMKDGI
NCU01479.1	G----------------PKRPGAQGQGGQEPPQGFDGGLMNYVIGTAVLFWTYKVLFPGN
FG06816.1 	Q----------------PKNERSG-------WLEAVQTGIAIGVTVWIAELFANPFSE--
AN4557.1  	Q----------------SDSPDFK-------TLDFKFDPASSLISTVIAYYIYRSIFPGD
          	                 ..                        :   :     . :  . 

MG07441.1 	NFMDLTMLGRAYLEVSRLPESEKAKLDKTVQNAYNLFSSNPPPGSQQAPSSGDGKEKKGR
NCU01479.1	D-----------------------------------------------------------
FG06816.1 	------------------------------------------------------------
AN4557.1  	N-----------------------------------------------------------
          	.                                                           

MG07441.1 	GSPPPQPDWKSSAVILSFIALWMLTSDSQGGKEITWQEFRSQYLDKGLVQKLTVS-GDTV
NCU01479.1	------------------------------SREITWQELRKNFLEKGLVEKLTVVK-DRV
FG06816.1 	-------------------------------KEITWQEFRKAFLDKGLVQKLVVVNGSQV
AN4557.1  	------------------------------SKDITWEEFRSKFLDKGLVERLTVTNRTRV
          	                              .::***:*:*. :*:****::*.* .   *

MG07441.1 	RVDLNPDA-TRAASGEMLTPR---VYFSIGSRDSFERKIEEAQAELGIPSSEQIPISYER
NCU01479.1	IVDLNKEAVRQMYPDSPAASPGFHYYFTIGSIDAFERRLDEAQAELGIPPAERIPVSYAN
FG06816.1 	RVELHPSAGATGENGQTGKRS---YVFTIGSVESFERKLENAQDELGIPSAERIPVSYEA
AN4557.1  	RVELNRDAVARTYPDSPAASPNFYYYFTVGSVESFERKLDQAQFELGIPTSERIPVSYVD
          	 *:*: .*      ..     .    *::** ::***::::** *****.:*:**:**  

MG07441.1 	P-SMIGSLIMSFAPTLLLVGLLAWISR--RGVGGGGGNPFKFGKSLAKPYTHESAVKVKF
NCU01479.1	EFSWGN-IILAFGPTLVLVGLLAYISRRGPGGAGGPGGMFGIGKSKAKMFNHDTAVKVKF
FG06816.1 	GGSTVGNLLLAFGPTLLFIGLILWTQRSMGGRGGAGGGMFNFGKSKAKKFNAESAVKVKF
AN4557.1  	EVPWMA-TVLSFGPTLLFMGGLFYLSRRAGSASAGRNGIFGIGKSRARRFNHETDVKIKF
          	  .     :::*.***:::* : : .*   . ... .. * :*** *: :. :: **:**

MG07441.1 	ADVAGADEAKLEIVEFVSFLKTPEKFVRLGAKIPRGAILSGPPGTGKTLLAKATAGESGV
NCU01479.1	SDVAGMDEAKVEIMEFVQFLKEPERFQKLGAKIPRGAILSGPPGTGKTLLAKATAGESQV
FG06816.1 	ADVAGLEEAKTEIMEFVSFLKQPEKFEKLGAKIPRGAILAGPPGTGKTLLAKATAGESGV
AN4557.1  	SDVAGMDEAKVEIMEFVSFLKSPERFQKLGAKIPRGAILSGPPGTGKTLLAKATAGESGV
          	:**** :*** **:***.*** **:* :***********:****************** *

MG07441.1 	PFFSVSGSEFVEMFVGVGASRVRDLFKEARKHAPCIIFIDEIDAIGKAR-DSGARFGGND
NCU01479.1	PFFSVSGSEFVEMFVGVGASRVRDLFATARKNAPCIIFIDEIDAIGRSRSDGGFRGGGND
FG06816.1 	PFFSVSGSEFVEMFVGVGPSRVRDLFAEGRKNAPCIIFIDEIDAIGRARQESGKGFGGND
AN4557.1  	PFFSVSGSEFVEMFVGVGPSRVRDLFANARKNTPCIIFIDEIDAIGKSRSAKNFSGG-ND
          	******************.*******  .**::*************::*.  .   * **

MG07441.1 	EREATLNQILTEMDGFNSNEQVVVLAGTNRPEILDKALMRPGRFDRHIHIDRPTMKGRAD
NCU01479.1	EREATLNQILTEMDGFNTTEQVVVLAGTNRPDILDKALMRPGRFDRHINIDRPTMKGRQD
FG06816.1 	EREATLNQILTEMDGFNTREQVVVLAGTNRADMLDKALMRPGRFDRHIFIDRPTMKGRQE
AN4557.1  	ERESTLNQILTEMDGFNTSDQVVVLAGTNRPDVLDKALMRPGRFDRHISIDRPTMDGRKQ
          	***:*************: :**********.::*************** ******.** :

MG07441.1 	IFKVHLARILTKEDFEYLCGRLSALTPGFAGADIANVVNEAALVA1ARDNADSVTMVHFE
NCU01479.1	IFKVHLAKIVTKEDIEFLTGRLAALTPGFAGADIANVVNEAALVA1ARANAETVEMIHFE
FG06816.1 	IFKVYLNKIVTNEDQEYLVGRLATLTPGFSGADIANVVNEAALIA1ARGNADDVKMDHFE
AN4557.1  	IFRVHLKKIVTKEDMDYLTGRLSALTPGFAGADIANCVNEAALVA1ARENAESVTMKHFE
          	**:*:* :*:*:** ::* ***::*****:****** ******:* ** **: * * ***

MG07441.1 	RAIERVIGGLERKSLVLGPEEKRTVAYHEAGHAVCGWYFQWADPLLKVSIIPRGSGALGY
NCU01479.1	QAIERVIGGLERKSLVLSPEEKRTVAYHEAGHAICGWFFQWADPLLKVSIIPRGQGALGY
FG06816.1 	RAIERVIGGLERKSLVLKPEEKKTVAYHEAGHAICGWFLEHADPLLKVSIIPRGQGALGY
AN4557.1  	RAIERVVGGLEKKSLVLSPEEKRTVAYHEAGHAICGWYFRWADPLLKVSIIPRGQGALGY
          	:*****:****:***** ****:**********:***::. *************.*****

MG07441.1 	AQYLPS-GDAYLMTVNQLMDRMAMTLGGRVSEELHFPTVTTGASDDFKKVTQMATTMVTQ
NCU01479.1	AQYLPS-GDAYLMNTKQLMDRMAMTLGGRVSEELHFPVVTTGASDDFKKVTRMARAMVTE
FG06816.1 	AQYLP--QDAYLMNTNQLMDRMAMTMGGRVSEELHFPTVTTGASDDFKKVSQMARNMVTQ
AN4557.1  	AQYLPANGDTYLMTANQMMDRMAMTLGGRVSEELHFDTVTSGASDDFNKVTRLATAMVTK
          	*****:. *:***..:*:*******:********** .**:******:**:::*  ***:

MG07441.1 	WGMSEKLGPLHFNQDPNQ-VQKPFAESTAQTIDAEVRRIVDEAYKKCRDLLTEKKAEVGI
NCU01479.1	WGMSEKVGMLHFD-DSAERFQKPFAESTAQAIDSEVRRIVDEAYKQCKDLLTAKKKEVGM
FG06816.1 	WGMSEIVGPVHFENDPNR-MQKPFAESTAQQIDLEVSRIVEEAYKRCKDLLTEKKNEVGL
AN4557.1  	FGMSPKLKYIYYEEDPSSQLHKPFSEETAKDIDIEVRRIVNEAYKQCRDLLTAKKKEVGL
          	:***  :  ::::.*.   .:***:*.**: ** ** ***:****:*:**** ** ***:

MG07441.1 	IAEELLRREQLTRDDIVRLLGPREWPDKEEFSKYFDGTNGGKSPLPTGENLRDPPAPPSP
NCU01479.1	VAEELLRKEVLSRDDLVRLLGPRQWPDKEEFSKYFHGERKSAPPPFPTENTDTPEERGPQ
FG06816.1 	IAQELLKKEVLVRDDMVRILGKRPFGDNEDFEKYFGGSKPESTPPPFPEETDSPNEPPTP
AN4557.1  	VAEELLAKEVLSRDDMVRLLGPREWPESGEFAKYFDG--KHGQTIAPPEPEVGPEAG---
          	:*:*** :* * ***:**:** * : :. :* *** *      .    *    *      

MG07441.1 	PAPEGGEGPVQPAAFRAIEDRR
NCU01479.1	PAMREEKADATGNKEGESKEQR
FG06816.1 	SP-----------AFKKVE---
AN4557.1  	PETRESPSS-------------
          	.      .
```
